# Benzylthiouracil-Induced Glomerulonephritis

**DOI:** 10.1155/2009/687285

**Published:** 2009-07-06

**Authors:** Sihem Trimeche Ajmi, Rim Braham, Sarra Toumi, Molka Chadli Chaieb, Amel Maaroufi, Koussay Ach, Larbi Chaieb

**Affiliations:** ^1^Department of Endocrinology, Farhat Hached Hospital, 4000 Sousse, Tunisia; ^2^Department of Internal Medicine, Sahloul Hospital, Sousse, Tunisia

## Abstract

Vasculitis is a rare complication of antithyroid drugs (ATDs). It was first described with Propylthiouracil (PTU). We report a new case of antineutrophil cytoplasmic antibody (ANCA) vasculitis with glomerulonephritis induced by Benzylthiouracile (BTU). A 50-year-old man with Graves disease treated with BTU developed general malaise and haematuria without skin rash or respiratory involvement. Laboratory data revealed acute renal failure with proteinuria and haematuria. An indirect immunofluorescence test for ANCA was positive, showing a perinuclear pattern with specificity antimyeloperoxidase (MPO). A renal biopsy was performed and revealed pauci-immune extracapillary glomerular nephropathy and necrotic vasculitis lesions. Based on these findings we concluded to the diagnosis of rapidly progressive glomerulonephritis associated with ANCA induced by BTU therapy. The drug was therefore discontinued and the patient was treated with steroids and immunosuppressive treatment during 3 months. Renal failure, proteinuria and haematuria significantly improved within 2 months. However, P-ANCA remained positive until 10 months after drug withdrawal. Thyroid function was kept within normal range using iodine solution. We demonstrated clearly that BTU may induce severe forms of vasculitis with glomerulonephritis. Thus, the ANCA must be measured when confronted to systemic manifestation during treatment.

## 1. Introduction

Antithyroid drugs (ATDs) such as Propylthiouracil (PTU) and Benzylthiouracil (BTU) are widely used for treatment of Graves disease. Commonly adverse effects related to the use of antithyroid drugs include agranulocytosis, cutaneous macular or papular skin rash, toxic hepatitis and induced lupus-like syndrome [1, 2]. ANCA positive vasculitis is a rare and severe complication of this treatment, described firstly with PTU [3], than with other ATD such as Carbimazole, Methimazole and recently Benzylthiouracil [4]. We report here a new case of Benzylthiouracil-induced ANCA positive vasculitis resulting in a necrotizing or crescentic glomerulonephritis.

## 2. Case Report

A 50-year-old man was admitted to the hospital because of general malaise and haematuria. He has a history of Graves disease diagnosed in 2004 and treated with Benzylthiouracile (Basdène) 300 mg/d for 8 months. On admission, his blood pressure was 120/70 mmHg and his pulse was regular at 108/min with no fever. On physical examination, the patient looked pale and his *conjunctivas were anaemic. *His thyroid gland was enlarged and there were no exophthalmia, skin rash or edema in lower extremities. Cardiovascular examination was normal. 

Urine analysis showed haematuria (4+) and proteinuria (2+). Laboratory data at admission showed: hemoglobin 9.4 g/dL, serum urea 19.7 mmol/L (normal range: 2.5–7.5 mmol/L ), serum creatinine 413 *μ*mol/L (normal range: 70–130 *μ*mol/L); it was 54 *μ*mol/L 8 months before, Proteinuria 1.1 g/d, haematuria 800 0000 red blood cells/mL, erythrocyte sedimentation rate 102 at 1 hour. Serum concentrations of CH50, C3 and C4 were in normal range. Thyroid function was normal with negative antithyroglobulin (anti-TG), antiperoxydase (anti-TPO) antibodies, and positive antithyrotropin receptor antibodies (TRAb) (range 40 UI/mL). Antinuclear and antiglomerular basement membrane antibodies were negative. An indirect immunofluorescence test for ANCA was positive, showing a perinuclear pattern with specificity antimyeloperoxydase (MPO). A renal biopsy was performed and revealed pauci-immune extracapillary glomerular nephropathy and necrotic vasculitis lesions. Based on these findings the diagnosis of rapidly progressive glomerulonephritis associated with ANCA induced by BTU therapy was strongly suggested. The drug was therefore discontinued and patient was treated with pulse of methyl prednisolone (500 mg/d for 3 days) followed by oral prednisolone (60 mg/d) and monthly intravenous pulses of cyclophosphamide during 3 months. Renal failure, proteinuria and haematuria significantly improved within 2 months. Serum creatinine level decreased to 84 *μ*mol/L and urine analysis revealed no proteinuria or haematuria. However, P-ANCA remained positive until 10 months after drug withdrawal. Thyroid function was kept within normal range using iodine solution.

## 3. Discussion

In this paper we reported a new case of BTU-induced ANCA positive vasculitis resulting in necrotizing glomerulonephritis. To our knowledge only four similar cases, with Benzylthiouracile, have been reported in literature [5–8]. ANCA vasculitis complicating antithyroid drugs was first reported in 1992 by Stankus and Johnsen in a patient who developed severe respiratory failure with PTU [3]. In 1993, Dolman et al. reported the detection of ANCA in serum of six patients who developed vasculitis during PTU treatment of hyperthyroidism [9]. In these patients renal function was normal with no proteinuria. The ANCAs are associated to systemic necrotizing vasculitis. The presenting symptoms of ATD induced ANCA vasculitis are variable including renal involvement, arthralgia, fever, skin involvement, respiratory tract involvement, myalgia or scleritis [4]. Our patient presented with clinical and biological disturbances related to renal failure with no medical history of pre-existing renal disease. Symptoms occurred 8 months after the beginning of treatment and there was no evidence of cutaneous vasculitis. ANCA positivity and renal biopsy which described pauci-immune extracapillary glomerular nephropathy concluded to the diagnosis of BTU induced vasculitis. Crescentic glomerulonephritis with ANCA positive vasculitis was firstly reported by Vogt in 1994, in 2 children receiving PTU [10]. Since then, many cases of ANCA positive vasculitis secondary to ATD have been described with mainly PTU [11, 12] and other ATD such as Carbimazole, Thiamazole [13, 14] and recently Benzylthiouracile [5–8]. Crescentic glomerulonephritis is histopathologically characterized by extracapillary proliferation and crescent formation in the majority of glomeruli with clinical syndrome of rapid deterioration in renal function occurring within few weeks or months [15]. Although immune complex was considered to be a possible cause of nephritis [15], its mechanism is still unknown. Griswold reported granular deposit of Immunoglobulin, complement in glomeruli on immunofluorescence, and concluded that vasculitis secondary to PTU is an immune complex disease [16]. Besides, it is known that thyroid antigens have been implicated in the pathogenesis of glomerulonephritis [17]. However, in our case, there were no significant deposit of immune complex and serum antithyroid antibodies (anti-TG and anti TPO) were negative. This kind of pauci-immune nephritis is compatible with ANCA induced rapidly progressive glomerulonephritis as reported by Kudoh [18]. Graves disease is the most common underlying thyroid disease. ANCA positive vasculitis has been less frequently reported in association with toxic multionodular goiter [19]. Severe adverse effects secondary to ATD such as agranulocytosis usually occur within weeks after initiation of therapy [20]. However, ANCA positivity seems to be correlated to duration of therapy, the risk is particularly important after 18 months of treatment [21]. The clinical significance of ANCA positivity is still controversial. It has been reported that 20% of patients with hyperthyroidism taking PTU were serum ANCA positive during the course of therapy but a small number of them develop vasculitis [9, 22]. Dolman demonstrated clearly the role of PTU in vasculitis in 6 patients with vasculitis disorders which disappeared with concomitant fall in ANCA titers after withdrawal of PTU [9]. The pathogenesis of ANCA mediated vasculitis is not well understood. It seems that Thionamide accumulates in neutrophils [23], binds to MPO inducing alteration of the cell structure [24] and then initiation of autoantibody formation. Falsely positive ANCA may be encountered in some patients without systemic manifestation of vasculitis. This phenomenon is explained by a cross reaction between antithyroid antibodies (anti-TRAb or anti-TG) and ANCA [25]. Approximately 90% of cases of ANCA positive vasculitis have occurred in association with PTU [4]. Only few cases of vasculitis induced by BTU have been reported in the literature involving principally the kidney and the respiratory tract [6–8]. In fact, the prevalence of PTU-treated patients is much higher than other antithyroid medication which is a matter of preference of each doctor and of availability of drugs. In Tunisia, all cases of ANCA-positive vasculitis descried to date were secondary to BTU, which is the main ATD used in practice. 

Treatment of ANCA positive vasculitis with renal involvement is controversial. After withdrawal of ATD, improvement of renal function was obtained in most reported cases [26, 27]. In the other cases, corticosteroid treatment and/or immunosuppressive treatment were given [28, 29]. Since renal involvement in ANCA positive vasculitis may be a cause of end-stage renal failure, our patient was treated with cyclophosphamide and prednisolone in addition to BTU withdrawal. Renal outcome was favorable but ANCA remained positive several months after withdrawal of BTU. Discontinuing ATD does not always result in fall in titer or disappearance of circulating ANCA. High MPO-ANCA titers in remission had still been observed in a substantial number of patients [30]. This may be a high risk of relapse implying long-term followup [31].

## 4. Conclusion

The association between Benzylthiouracile and ANCA vasculitis is now well documented. Although its rare occurrence, attention must be given to systemic symptoms such as skin lesion, haematuria or proteinuria. The ANCA must be measured when confronted to systemic manifestation during treatment.
